# Accessibility of malaria commodities in Geita District Council, mainland Tanzania: the experiences from healthcare providers and clients

**DOI:** 10.1080/20523211.2024.2308611

**Published:** 2024-02-07

**Authors:** Anna David, Omary Swalehe, Jean D’ Amour Habagusenga, Stany Banzimana, Domina Asingizwe, Frank Chacky, Fabrizio Molteni

**Affiliations:** a EAC Regional Centre of Excellence for Vaccines, Immunization, and Health Supply Chain Management, College of Medicine and Health Sciences, University of Rwanda, Kigali, Rwanda; b Department of Business Studies, Mzumbe University, Dar es salaam, Tanzania; c National Malaria Control Programme, Ministry of Health, Dodoma, Tanzania; d Swiss Tropical and Public Health Institute, Dar es Salaam, Tanzania

**Keywords:** Accessibility, factors, malaria commodities, Tanzania

## Abstract

**Background:**

Access to essential malaria commodities is a cornerstone in malaria control. However optimal availability and access to essential malaria commodities remain a challenge in Tanzania. Therefore, this study aimed to explore the factors affecting the accessibility of malaria commodities in Tanzania.

**Methods:**

This was a mixed-method cross-sectional study using both quantitative and qualitative approaches. Data were collected between February and March 2023 from health facilities, health facility staff, and patients.

**Results:**

Availability of malaria commodities in government health facilities was 100% for all items while in the private and faith-based facilities, this ranged from 10% to 80%. The reasons for stockouts in Government facilities were related to delayed and inadequate quantity delivery while in private facilities the main reason was the lack of cash for procurement. Both private facilities’ clients and healthcare providers concurred that most people do not access complete treatment due to the high costs of prescribed medicines and poor stocking levels.

**Conclusion:**

The availability, hence the accessibility, of malaria commodities in private and faith-based health facilities is still sub-optimal. Logistic management needs to be improved to eliminate stockouts and malaria commodities high costs need a permanent solution.

## Introduction

Malaria remains a significant public health concern in the African region due to its high transmission, morbidity, and mortality rates (WHO, 2022). In 2021, the World Health Organization estimated 619,000 malaria deaths globally and among them 96% of malaria deaths (593.000) were occurring in Africa (WHO, 2022). In addition, 247 million malaria cases globally were reported of which 95% (234 million) were from Africa (WHO, 2022). Several malaria vector control and case management initiatives have been implemented in Sub-Saharan Africa to prevent and mitigate the effects of malaria infection (Oladipo et al., 2022; Orok et al., 2021). Despite making good progress in reducing malaria incidence, these initiatives have been unable to meet the Global Technical Strategy Milestones of reducing malaria mortality and morbidity globally by at least 40% by 2020, which may delay achieving the global strategy target of reducing malaria incidence and mortality rates by at least 90% by 2030 (WHO, 2015; WHO, 2022).

In Tanzania, Malaria Indicator Surveys reported significant progress in reducing malaria prevalence by more than 50% from an average of 18.1% in 2008 to 8.1% in 2022 (Tanzania Commission for AIDS (TACAIDS), et al., 2008; Ministry of Health – Tanzania Mainland, et al., 2022). However, malaria transmission risk remains moderate to high in approximately one-third of the country [6]. The burden is higher in North West and South East areas of Tanzania with persistently detected high transmission intensity (Ministry of Health – Tanzania Mainland, 2022; Ministry of Health –Tanzania Mainland, et al., 2023).

To effectively prevent, diagnose, and treat malaria, it is essential to ensure the accessibility of malaria commodities in healthcare facilities throughout the country (Demessie et al., 2020; WHO, 2022), however, in Sub-Saharan Africa, challenges in the accessibility of malaria commodities in public health facilities still exist (Demessie et al., 2020; Ooms et al., 2020; Uwizeyimana et al., 2021; WHO, 2022). Evidence from Ghana, Kenya, and Uganda (Masters et al., 2014), shows that due to frequent stockouts in public healthcare facilities, users are forced to shift to the private health sector to access treatment.

Numerous reforms and initiatives introduced in Tanzania over the past two decades, including new technologies and infrastructure, have improved the availability of health commodities (Githendu et al., 2020). Despite this fact, shortages of essential medicines, including malaria commodities, have been reported in public and private health facilities, especially in remote areas (Martin et al., 2023; Michael & Mkunde, 2017; Worges et al., 2022). While considerable progress in distributing malaria commodities in Tanzania has been observed over time, many medicines and other commodities required by health facilities are still insufficiently supplied and utilised (Emerson et al., 2023; Exavery et al., 2014; Hasselback et al., 2014; Martin et al., 2023; Michael & Mkunde, 2017; Worges et al., 2022). Therefore, the purpose of this study was to investigate the determinants influencing the accessibility of malaria commodities in health facilities in Geita District Council, Tanzania.

## Methods

### Study setting

This study was conducted in health facilities of Geita District Council, north-western Tanzania. Geita District Council was selected purposefully among the councils with high malaria prevalence (38.5%) based on the School Malaria Parasite Survey 2021. Based on the Geita Regional Comprehensive Council Health Plan for the fiscal year 2021/2022, the total number of health facilities providing healthcare services was 62, comprising 48 public, 11 private, and 3 managed by faith-based organisations. Based on the levels, there was 1 tertiary level hospital, 8 secondary level health centres, and 53 primary level dispensaries.

### Study design, population, and sampling

This is a mixed method study design whereby the quantitative section used the standard World Health Organization/Health Action International Organization (WHO/HAI) approach for measuring the availability and prices of malaria commodities and the qualitative section consisted in interviewing people in one-on-one conversations using key informant and in depth interview guide. Data were collected from health facilities, healthcare providers, and clients who attended that particular health facility to seek malaria services on the day of the interview, and review of health commodity management tools.

For the quantitative part, 58 health facilities of Geita District Council providing malaria services, 48 public, 8 private, and 2 faith-based organisation health facilities, were considered to determine the availability and prices of malaria commodities. Pre-testing was performed in two dispensaries. The other two facilities were not fully operational and did not provide malaria services during the research period and, therefore, were excluded from the study.

For the qualitative, 49 interviews were conducted in 14 out of 58 visited health facilities: 6 government, 6 private, and 2 faith-based. The respondents were purposefully chosen. The interviews included 28 key informant interviews (KII) with healthcare providers, 2 per health facility, including the in-charge and the person responsible for inventory management. Healthcare providers were categorised into six professions, namely pharmaceutical personnel (5), nurses (11) including enrolled nurses, assistant nurses and assistant nursing officers, clinical officers (7), medical officers (3), procurement officer (1), as well as a clinical tracker (1). In addition, 21 in-depth interviews were conducted with clients seeking malaria treatment-related services in the same 14 health facilities out of 58 visited. The sample size for the qualitative part for both healthcare providers and the clients was determined based on saturation. Finally, store ledger malaria commodities’ records were reviewed and physical verification of the stock was done.

### Data collection instruments and measurements

The data collection tools are adapted from the Logistics System Assessment Tool (LSAT) developed by USAID | DELIVER PROJECT. It consisted of a key informant interview guide for healthcare providers, which collected information on the availability and affordability of malaria commodities and their acceptability in implementing the strategy of free-of-charge malaria commodities. The interview guide for clients receiving malaria services collected information on affordability, physical accessibility to the point of care, and acceptability of the services received by the clients. The checklist collected information on malaria commodity availability and price as per the WHO/HAI methodology. The informed consent form explained the significance of the study, confidentiality, and voluntary participation in the study to participants.

The in-depth interview guide was used to provide detailed information on the following four main variables: availability: having malaria commodities continuously at public or private health facilities, on the day of the visit and during the review period which was from January to December 2022. Acceptability: the extent to which the client is comfortable with the malaria service provided and the immutable characteristics of the provider. Affordability: having a cost that is perceived not to be too high, products sold at affordable prices; and geographical accessibility − which is determined by how easily the client can physically reach the health facility.

### Data collection procedures

A team of four research assistants were adequately trained on research tools and procedures. The training was followed by pretesting the tools in two facilities in Geita District Council. Geita Region and Geita District Council authorities were informed about the study and communicated formally to the health service providers the arrangements and the date of the visit. Consent to conduct an interview including recording and taking notes during the discussions was obtained before the start of the interview and the confidentiality of all informant’s responses was ensured.

Audio devices were used to record the information. KII took place at the office of the health facility in charge and other areas directed by the in charge which provided privacy, and took 15–25 min depending on the informant willingness to speak longer.

For malaria commodity availability and prices, the pre-tested checklist were used to collect information from health commodity management tools like store ledgers and invoices. The content of data collection tools was reviewed by the data collection supervisor for completeness, clarity, and readability. Also, there was close follow-up supervision to data collectors and checking the completeness of data on a daily basis. To ensure consistency, the tools were structured in a way that some of the requested information was counter-checking the consistency and quality of other provided information.

### Data management and analysis

The quantitative data entry, cleaning, and analysis were performed using STATA version 15.1. Mean per cent availability and standard deviation were generated as per the standard WHO/HAI methodology for analysis of the availability of health commodities.

The audio-recorded qualitative data were transcribed and translated into English. The analysis was conducted using deductive content analysis. The researcher focused on interpreting and understanding the text, categorised and coded the text, organised coded text into themes and elucidated relationships between themes and did the final description of the findings. The first author generated the initial list of codes. The second author reviewed and revised the list as appropriate. The whole team then reviewed, revised, and agreed on the final list of codes, as well as the categories and themes.

## Results

### Characteristics of health facilities visited

Among visited health facilities 49 (84%) were dispensaries, 8 (14%) were health centres, and 1 (2%) was hospital. The majority of health facilities visited were owned by the government 48 (83%), while the remaining 8 (14%) and 2 (3%) were owned by the private sector and faith-based organisations respectively.

### Availability of malaria commodities at health facilities

All malaria commodities reviewed in the study were available on the day of the visit in all government health facilities. Availability of malaria commodities in private-for-profit and faith-based facilities ranged from 10% to 80% for different items (Table 1).

**Table 1.** Status for different malaria commodities availability in health facilities.

Commodity type	Government (*n* = 48)	Private and FBO (*n* = 10)	Overall (*N* = 58)
ALu 1 × 6	48 (100%)	2 (20%)	50 (86.2%)
ALU 2 × 6	48 (100%)	1 (10%)	49 (84.5%)
ALu 3 × 6	48 (100%)	1 (10%)	49 (84.5%)
ALu 4 × 6	48 (100%)	6 (60%)	54 (93.1%)
MRDT	48 (100%)	3 (30%)	51 (87.9%)
Artesunate inj	48 (100%)	3 (30%)	51 (87.9%)
SP	48 (100%)	6 (60%)	54 (93.1%)
LLIN	48 (100%)	8 (80%)	56 (96.5%)

Notes: ALu = Artemether-Lumefantrine; MRDT = malaria rapid diagnostic test; Inj = Injectable; SP = sulfadoxine-pyrimethamine; LLIN = long lasting insecticide treated net.

However, in the 12 months reviewed, the stock of malaria commodities in the visited health facilities was observed in different periods ranging from a minimum of 7 days and a maximum of 360 days (Table 2).

**Table 2.** The summary statistics on stock out of malaria commodities.

Commodity	Proportion of Stock out	Total Health Facilities	Mean days of stock out	SD	Min	Max
ALu 1 × 6	32 (55.1)	58	95.8	101.7	28	360
ALU 2 × 6	25 (43.1)	58	109.1	123.0	13	360
ALu 3 × 6	18 (31.0)	58	136.7	134.6	30	360
ALu 4 × 6	17 (29.3)	58	68.1	84.2	7	360
MRDT	10 (17.9)	56	132.2	141.0	6	360
Artesunate inj	11 (19.6)	56	94.1	95.9	12	240
SP	17 (32.69)	52	75.1	81.1	10	360
LLIN	7 (14.0)	50	78.9	54.0	15	180

Notes: ALu = Artemether-Lumefantrine; MRDT = malaria rapid diagnostic test; Inj = Injectable; SP = sulfadoxine-pyrimethamine; LLIN = long lasting insecticide treated net.

These quantitative findings were complimented by the majority of respondents (healthcare providers) during key informant interviews. Ten out of 11 healthcare providers at private health facilities, 11 out of 13 from government health facilities, and 2 out of 4 interviewed at faith-based organisation health facilities reported that there are no challenges faced on malaria commodity availability. One respondent stated:

There is 100% availability of malaria commodities, no any scarcity but we had excess which we decided to share to the nearly facilities. This was due to the fact that we were supplied with more health commodities than our needs. **(Healthcare provider from Government health facility 5)**

However, 2 out of 13 healthcare providers from government health facilities, faith-based organisation (1 out of 4 healthcare providers), and private health facilities (1 out of 11 healthcare providers) mentioned that challenges still exist in the availability of malaria commodities because sometimes they run out of stock to the extent of going to borrow from other health facilities. The issues pointed out to be associated with delays in dispatching commodities as per order/delay in malaria commodities supply and the high price of malaria commodities due to scarcity in the market. This was explained as follows:

In general, the availability is good, but few months ago artesunate injection were finished, there were some difficulties but we immediately ordered. As we speak now, there is no difficulty, it was only a short period. **(Healthcare provider from Government health facility 1)**

My opinion is that, Medical Store Department (MSD) should increase the availability of all medicine because there is a challenge of not receiving some of the ordered malaria commodities in the scheduled period. **(Healthcare provider from Government health facility 6)**

Better availability profile of malaria commodities in government health facilities was attributed to the government free of cost policy. However, providers claimed that, in case of less stock, they had to use health facility funds to purchase medicines and tests from accredited prime vendors. One participant indicated:

All the costs to procure malaria commodities are incurred by the ministry of health from the direct fund sourced from the Ministry of Finance and Planning. **(Healthcare provider from Government health facility 2)**

Private health facilities, including faith-based facilities, generally receive funds for procurement directly from the managing authority or, occasionally, purchase medical supply directly using patients’ user fees. One of the key informants expressed:

We buy our medicines and we pay from money which is obtained from patients who are using cash and health insurance cards. **(Healthcare provider from private health facility 1)**

### Affordability of malaria commodities

The price of malaria commodities in the private sector was varying from one private health facility to another. Prices ranged from 4.35$ for the Artesunate injection to as low as 0.43$ for the Alu 1 × 6. Table 3 provides details.

**Table 3.** Range of the malaria commodities prices in private health facilities.

Commodity	Mean	Standard deviation	Minimum	Maximum
ALu 1 × 6	$ 0.96	$ 0.44	$ 0.43	$ 1.74
ALU 2 × 6	$ 1.04	$ 0.35	$ 0.65	$ 1.74
ALu 3 × 6	$ 1.24	$ 0.34	$ 0.65	$ 1.74
ALu 4 × 6	$ 1.31	$ 0.57	$ 0.87	$ 2.17
MRDT	$ 0.87	–	$ 0.87	$ 0.87
Artesunate injection	$ 3.24	$ 0.93	$ 2.17	$ 4.35
SP	$ 0.83	$ 0.10	$ 0.65	$ 0.87
LLIN	–	–	–	–

Notes: ALu = Artemether-Lumefantrine; MRDT = malaria rapid diagnostic test; Inj = Injectable; SP = sulfadoxine-pyrimethamine; LLIN = long lasting insecticide treated net.

Key informants from the private health sector and faith-based health facilities reiterated that they do not get any subsidy or free-of-charge items from the government, hence patients are required to pay for the services and commodities provided in their facilities; these respondents were all from private health facilities (11 out of 11) and faith-based organisation health facilities (2 out of 4). One participant indicated:

… they pay for consultation fee and commodities, only mosquito nets are free. **(Healthcare provider from FBO health facility 1)**

… the patient is required to pay for medicine and treatment. (**Healthcare provider from private health facility 1)**

Moreover, 2 healthcare providers out of 13 interviewed in government health facilities and 1 out of 4 healthcare providers from faith-based health facilities pointed out that usually patient pays for user fees which is 3000 Tshs which is approximately to 1.3 USD, per person. One participant indicated:

Customers pay only 3000 Tshs for the user fee at outpatient department. **(Healthcare provider from Government health facility 6)**

In addition, healthcare providers from the private health sector confirmed that patients are required to pay cash for the services provided. Some pay through health insurance, however, very few customers’ benefit from health insurance. Users with very low income are not able to afford the entire costs for the services, hence they are not dispensed with either all the prescribed medicines or given short treatment course. This was reported by three respondents interviewed in private health facilities, one respondent interviewed in government health facilities and two interviewees contacted from faith-based health facilities. This was explained as follows:

They pay through cash. Some people are able to pay and other fail to pay because of lack of money. **(Health care provider at Dispensary 2 – faith based organization)**

They pay cash for medicines, for those who can pay for it. Those who don’t have money at that time we advise them to come back and collect the medicine when they get money because it is important for their health. **(Health care provider at Dispensary 2 – faith based organization)**

They pay with cash. Some of them are able to pay for the cost while others are given loan of services and after getting money they come back and pay. **(Health care provider at Dispensary 1 – private health facility)**

Furthermore, about three quarter of client’s interviewed (16/21 equally divided in attending public and private sector) did not had any challenge in accessing malaria commodities. However, the remaining quarter (5/21) failed to obtain the prescribed commodities due to the high cost. Among them, a few public service clients reported that they have been prescribed medicines to be purchased in the private pharmacies. The costs incurred by the private sector users ranged from 27,000 TZS (11 USD) to 80,000 TZS (24 USD). This was explained as follows:

Yes, we pay 27,000 TZS for all service, I was given enough medicine. **(Client at Government health facility 4)**

I'm not sure why malaria treatment service is so expensive; perhaps it's because this is a private hospital. **(Client at private health facility 2)**

### Accessibility of malaria commodities

The time spent by the clients/users to reach the health facility was also mentioned as an obstacle to access test and treatment services in government health facilities. One participant indicated:

I live far, it takes 1 hour walking from home to here. **(Client at Government health facility 4)**

However, the major obstacle for continuous access to malaria commodities in public service was reported to be the malfunctioning of the supply chain system to deliver the needed number of commodities in time, hence patients are sometimes instructed to go to a pharmacy and purchase medications or other malaria-related commodities suggested by their doctor. In the private sector, the main complaints were the inability of clients to pay for the services even though a large number of people used to attend their facilities due to proximity from their homes. This was explained as follows:

Sometimes when they say there is no medicine for the child, they tell you to go and buy it. Also, when patients arrive, they should have more testing equipment so that we can be served faster. **(Client at Government health facility 4)**

Health providers should make sure that the facility is equipped with medicines and test all the time. (**Client at Government health facility 3)**

### Healthcare providers acceptability in implementing free-of-charge malaria commodity policy

Approximately half (6 out of 13) of healthcare providers in government health facilities, 3 out of 11 in private health facilities and 2 out of 4 in faith-based health facilities were aware that malaria policy is based on free-of-charge services. Other providers reported that services should be free of charge for special groups, including children under the age of five, pregnant women, and the elderly. One participant indicated:

I understand that the malaria commodities are free at the governmental facilities, but we, private health sector we buy medicines and we cannot distribute them for free. **(Healthcare provider at private health facility 5)**

Healthcare provider generally know the aim of the ‘malaria commodities free strategy’. One participant indicated:

Malaria commodities are very expensive so the lower-class people wouldn’t get access to it easily. The government is buying the commodities so everyone could get the commodity. **(Healthcare provider at Government health facility 4)**

The majority of healthcare providers (16/28) agreed with the free-of-charge strategy and suggested that the government should also supply no-cost commodities to private-for-profit and FBO facilities. This was explained as follows:

There is no need to change this policy, I agree that it should be free because there are people that cannot afford. **(Healthcare provider at FBO facility 1)**

Government should bring the same strategy to private sector, as it is done with HIV/AIDS and Mosquito nets, so they can provide free of charge services. **(Healthcare provider at private health facility 2)**

Other healthcare providers from both government and private sectors suggested changes to malaria free services strategy. One participant indicated:

There is need to provide good service and to do it we need to increase the facility income. Patient who can afford should pay for services while others that cannot afford should be exempted. **(Healthcare provider at Government health facility 1)**

One provider reported that a free-of-charge policy might increase commodities abusive behaviours from people attending services even when it is not needed. One participant indicated:

Challenge of free malaria commodities is, few patients do not complete the dose because they know that he/she can get it again for free. **(Healthcare provider from Government health facility 1)**

### Client’s acceptability in malaria service provision

The majority of participants (clients 15/21), 10 respondents who were interviewed in government health facilities, and five participants who were contacted for interviews in private health facilities, were satisfied with malaria services they received from respective health providers. Some of the clients interviewed expressed:

I have been treated properly. **(Client at Government health facility 4)**

This health facility is near to my place, services are good and the providers were keen to welcome us. (**Client at private health facility 2)**

However, a few participants (3/21) were not satisfied with the malaria services they received, mainly because of the high price, availability of medicines, and long waiting times. Some of the clients interviewed expressed:

I am not satisfied with the services, am forced to reduce my child's treatment services due to the prices. **(Client at private health facility 2)**

There are challenges we are facing; we are waiting for a long time see the doctor in order to get the services. **(Client at Government health facility 4)**

There are challenges in receiving services because the testing equipment and medicines are not always available and sometimes the testing equipment was not working. **(Client at Government health facility 4)**

… when we arrive, they should have more testing equipment so that they can serve us faster. (**Client at Government health facility 4)**

## Discussion

This study was conducted to explore the factors affecting the accessibility of malaria commodities in Geita district Council, Tanzania and this included the following main variables: availability, affordability, physical accessibility, and acceptability. The discussion is therefore, structured following these main variables assessed in this study.

### Availability of malaria commodities

This study revealed optimal availability of malaria commodities on the day of visit in government health facilities, compared to the poor stocking level in the private and faith-based ones. However, malaria commodities stockouts were frequently recorded in the review period in all health facility irrespectively from types and ownership, though stockouts in private health facilities were more frequent. This finding is similar to the study conducted in Tanzania, which found improved availability of malaria commodities in the government health facilities, and the availability in the private sector remain sub-optimal (Michael & Mkunde, 2017). The reported findings also corroborate with the ones in a study conducted in Mozambique which revealed stockout of malaria commodities due to logistics and infrastructure issues (Davlantes et al., 2017). The better availability profile of malaria commodities in the government health facilities may be related to the adequate stock level of free of charge malaria commodities from the distributor, Medical Store Department, while the high frequency of stockouts in the private sector may be associated with inadequate funding for the procurement of medical supplies from the managing authorities.

Frequent stockout of malaria commodities has impacted malaria service by disrupting both distribution of long-lasting insecticide treated nets and access to testing and treatment of malaria which have effect in both preventive and curative services and ultimately on health outcome. If not addressed timely, the unavailability of essential commodities may impact the countries’ ability to achieve global malaria elimination goals.

### Affordability of malaria commodities

The study demonstrated that most clients afford to receive malaria services in government health facilities because they do not pay for malaria commodity, even though they pay for the consultation fees. This finding corroborates with commodity security framework for planning 2021 (Tetteh, 2021). This framework, based on studies reporting availability of health commodities in low-mid income countries (LMICs), recommends that steady supply of health commodities in poor resource settings can be addressed only by continuous provision of financial capitals. The implementation of malaria commodity free-of-charge strategy in the public health facilities needs continues disbursement of financial resources from government and development partners. Over the last decades, the availability of free of charge policy has increased the access to malaria diagnoses and treatment services and reduced inequality in healthcare delivery.

Nevertheless, the study findings revealed that clients pay for malaria commodities in private and faith-based health facilities. As a consequence, the high price of services affected the access to commodities for those who are not able to pay. In the event of stockouts of malaria commodities in public health facilities, the clients are forced to pay for purchasing them in the private sector. Low affordability is another aspect that limits access to services of a large portion of the population with no insurance and low income.

The above findings are similar to those of a 2018 study conducted in Ethiopia which revealed that low monthly income and not having a health insurance coverage are among the factors limiting malaria case management services (Tiruneh et al., 2018). Failure to reach the poorest segments of the populations of developing countries is among the public health threats. Interventions designed to provide affordable curative services to the lowest wealthy people that are mostly affected by malaria are of paramount importance.

### Healthcare provider’s acceptability on implementation of free of charge malaria commodity policy

The study indicated that the majority of healthcare providers in both public and private health facilities understand and accept the implementation of the strategy of free of charge malaria commodity in public health facilities. Other service providers declared to be aware of the policy but, in reality, they were misunderstanding it with other health commodity subsidy strategy. A qualitative study conducted in Malawi revealed similar findings (Wilhelm et al., 2016). The study revealed that for improved implementation of the strategy, its stakeholders’ acceptance grew stronger over time as understanding of the intervention improved and supported by early inclusion during the design and implementation process. The inadequate understanding of the strategy may be due to limited advocacy to the stakeholders. This may affect the access to malaria commodities because healthcare providers readiness influences the use of these services and improves the health system performance regarding malaria control programme milestones.

### Client’s acceptability to malaria commodities services

The current study revealed a mix of satisfaction and dissatisfaction among participants about malaria services they received. This is similar to a study conducted in Nigeria which revealed that although a fair proportion of respondents were satisfied with the preventive malaria services, most were dissatisfied due to the high costs of accessing care (Obagha et al., 2020). Furthermore, a 2022 study in Ethiopia revealed that long waiting time at outpatient departments affected the willingness of the clients to access the services (Biya et al., 2022). The study reported reasons of service dissatisfaction are attributed to challenges in the availability of medicines, long waiting time, and high price of malaria commodities. Patient service delivery satisfaction is a basic factor to access to services and it might affect timely, efficient, and patient-centred delivery of quality health care.

## Conclusion

This study aimed to explore the factors affecting accessibility of malaria commodities in the high malaria risk in Geita District Council, Geita region, Tanzania. It revealed the pattern of client’s malaria commodities’ affordability, accessibility, and acceptability. The study reported the inadequate availability of malaria commodities in private and faith-based health facilities. The situation in public healthcare services was comparatively better but still affected by period of commodities stockouts. Therefore, strategies should be put in place to reduce waiting time especially in public health service to increase acceptability. In the same line, improving knowledge of healthcare providers about the current malaria policy and strategies to improve their readiness is important.

## Data Availability

Datasets used during the study are available from the corresponding author on reasonable request.
